# Bällchen is required for self-renewal of germline stem cells in *Drosophila melanogaster*

**DOI:** 10.1242/bio.20147690

**Published:** 2014-05-29

**Authors:** Bettina Herzig, Toma A. Yakulov, Kathrin Klinge, Ufuk Günesdogan, Herbert Jäckle, Alf Herzig

**Affiliations:** Department of Molecular Developmental Biology, Max-Planck-Institut für Biophysikalische Chemie, Am Fassberg 11, 37077 Göttingen, Germany; * Present address: Renal Division, University Hospital Freiburg, Hugstetter Strasse 55, 79106 Freiburg, Germany.; ‡ Present address: Wellcome Trust/Cancer Research UK Gurdon Institute, University of Cambridge, Tennis Court Road, Cambridge CB2 1QN, UK.; § Present address: Department of Cellular Microbiology, Max-Planck-Institut für Infektionsbiologie, Charitéplatz 1, 10117 Berlin, Germany.

**Keywords:** Bällchen, *Drosophila*, Germline stem cells

## Abstract

Self-renewing stem cells are pools of undifferentiated cells, which are maintained in cellular niche environments by distinct tissue-specific signalling pathways. In *Drosophila melanogaster*, female germline stem cells (GSCs) are maintained in a somatic niche of the gonads by BMP signalling. Here we report a novel function of the *Drosophila* kinase Bällchen (BALL), showing that its cell autonomous role is to maintain the self-renewing capacity of female GSCs independent of BMP signalling. *ball* mutant GSCs are eliminated from the niche and subsequently differentiate into mature eggs, indicating that BALL is largely dispensable for differentiation. Similar to female GSCs, BALL is required to maintain self-renewal of male GSCs, suggesting a tissue independent requirement of BALL for self-renewal of germline stem cells.

## INTRODUCTION

Tissue specific stem cell populations are actively maintained as a source for distinct cell types required for growth, development and regeneration in the adult organism (Kohlmaier and Edgar, 2008; Weissman et al., 2001). To suppress their differentiation, stem cells require extracellular signalling cues which derive from a distinct cellular microenvironment called the stem cell niche (Losick et al., 2011). In the female ovary of *Drosophila melanogaster*, germline stem cells (GSCs) generate asymmetric cell fates with each cell division (Li and Xie, 2005; Morrison and Spradling, 2008). One daughter cell maintains the stem cell properties of the parent GSC, whereas the other daughter cell differentiates into a cystoblast. The cystoblast then develops into a germline cyst that includes a single cell that later becomes the oocyte. The germline cyst is surrounded by follicular epithelial cells, which are derived from follicle stem cells (FSCs). Similarly, the GSCs of the male germline also self-renew and differentiate into gonialblasts, which then give rise to sperm.

The stem cell niche of the ovary contains cap cells that secrete the *decapentaplegic* (DPP) ligand that activates the BMP pathway in GSCs. The resultant BMP signalling leads to phosphorylation of the transcription factor *Mothers against Dpp* (MAD), which represses the expression of the differentiation factor *bag of marbles* (BAM) (Chen and McKearin, 2003; Song et al., 2004). In the male germline, the Jak/Stat signalling pathway plays a major role for GSC self-renewal (Kiger et al., 2001). However, its cell autonomous function in GSCs is restricted to orienting the division plane of GSCs relative to the niche cells (Leatherman and Dinardo, 2010). The function of Jak/Stat signalling for GSC self-renewal primarily derives from activating the secretion of the BMP ligand *glass bottom boat* (GBB) in male somatic stem cells, which in turn activates the BMP pathway in GSCs (Kawase et al., 2004).

The progenitors of GSCs, which are called primordial germ cells (PGCs), also utilise BMP signalling through DPP to repress BAM in the larval ovary (Gilboa and Lehmann, 2004). Maintenance of FSCs in the ovary not only requires DPP signalling but also *hedgehog* and *wingless* pathway activity (for a review, see Kirilly and Xie, 2007). Thus, BMP signalling contributes both directly and indirectly to stem cell maintenance in various stem cell populations, but it acts in conjunction with other different external signals to suppress stem cell differentiation. However, common stem cell autonomous components that maintain stem cell properties and prevent differentiation have remained unknown.

Here we report that the protein kinase encoded by the *Drosophila melanogaster* gene *bällchen* (*ball*, also known as *Nhk-1*) has a common role in maintaining self-renewal of stem cell populations. *ball* protein (BALL) is orthologous to the Vaccinia-related Kinases (VRKs) of vertebrates and most closely related to VRK-1 (Aihara et al., 2004). VRKs are found in all metazoan species ranging from worms to humans. VRKs of different species were found to phosphorylate the Barrier-to-Autointegration Factor (BAF) (Bengtsson and Wilson, 2006; Gorjánácz et al., 2007; Lancaster et al., 2007; Nichols et al., 2006), which is involved in the assembly of the nuclear lamina in *Caenorhabditis elegans* (Gorjánácz et al., 2007) and the organisation of chromatin in the nucleus (Margalit et al., 2007). Moreover, hypomorphic mutations in *Drosophila ball* cause aberrant chromatin organisation in the oocyte nucleus and an altered pattern of histone modifications (Ivanovska et al., 2005). The analysis of *ball* null mutants revealed defects in proliferating tissues of the larvae, including the brain and imaginal discs (Cullen et al., 2005).

We have used systemic null mutants and mosaic analyses to characterise the function of BALL in both progenitor cells and niche-dependent stem cells. We found that BALL is required to maintain self-renewal of stem cells, which suggests that the previously described defects in proliferating tissues of *ball* mutant animals is caused by the premature or unscheduled differentiation of progenitor cells rather than a general function of BALL for cellular proliferation.

## RESULTS

### BALL is essential to maintain the larval germline

In order to assess the function of *ball* in proliferating tissue and in stem cells, we generated a null allele of *ball* (*ball^2^*; supplementary material Fig. S1). *ball^2^* homozygotes (hereafter referred to as *ball^2^* mutants) die during the pupal stage, confirming previous results described for other *ball* null alleles (Cullen et al., 2005). *ball^2^* mutants already show severe morphological defects by the end of larval development which include considerably reduced gonads in both sexes, the absence of imaginal discs and severely diminished larval brains. This mutant phenotype is solely due to the *ball^2^* mutation, as a genomic *ball* transgene rescued the mutants to produce viable and fertile adults (supplementary material Fig. S1).

We explored the function of BALL in developing male and female gonads. Growth of larval testes relies on asymmetric, niche-supported divisions of GSCs, whereas growth of larval ovaries relies on symmetric divisions of primordial germ cells (PGCs) (Dansereau and Lasko, 2008). Both larval cell types are derived from embryonic PGCs, which reside in the primitive gonads of embryos. In order to establish whether the initial number of PGCs was affected in *ball^2^* mutant embryos, we counted their number in embryonic gonads. *ball^2^* mutants contained on average 10.2 PGCs (SD = 1.8, *n* = 25 gonads) which was not significantly different from the number of PGCs observed in wild type control embryos (11.0 PGCs; SD = 1.4, *n* = 27 gonads). Therefore, the smaller size of larval gonads in *ball^2^* mutants is not caused by a reduced number of embryonic PGCs.

**Fig. 1.**
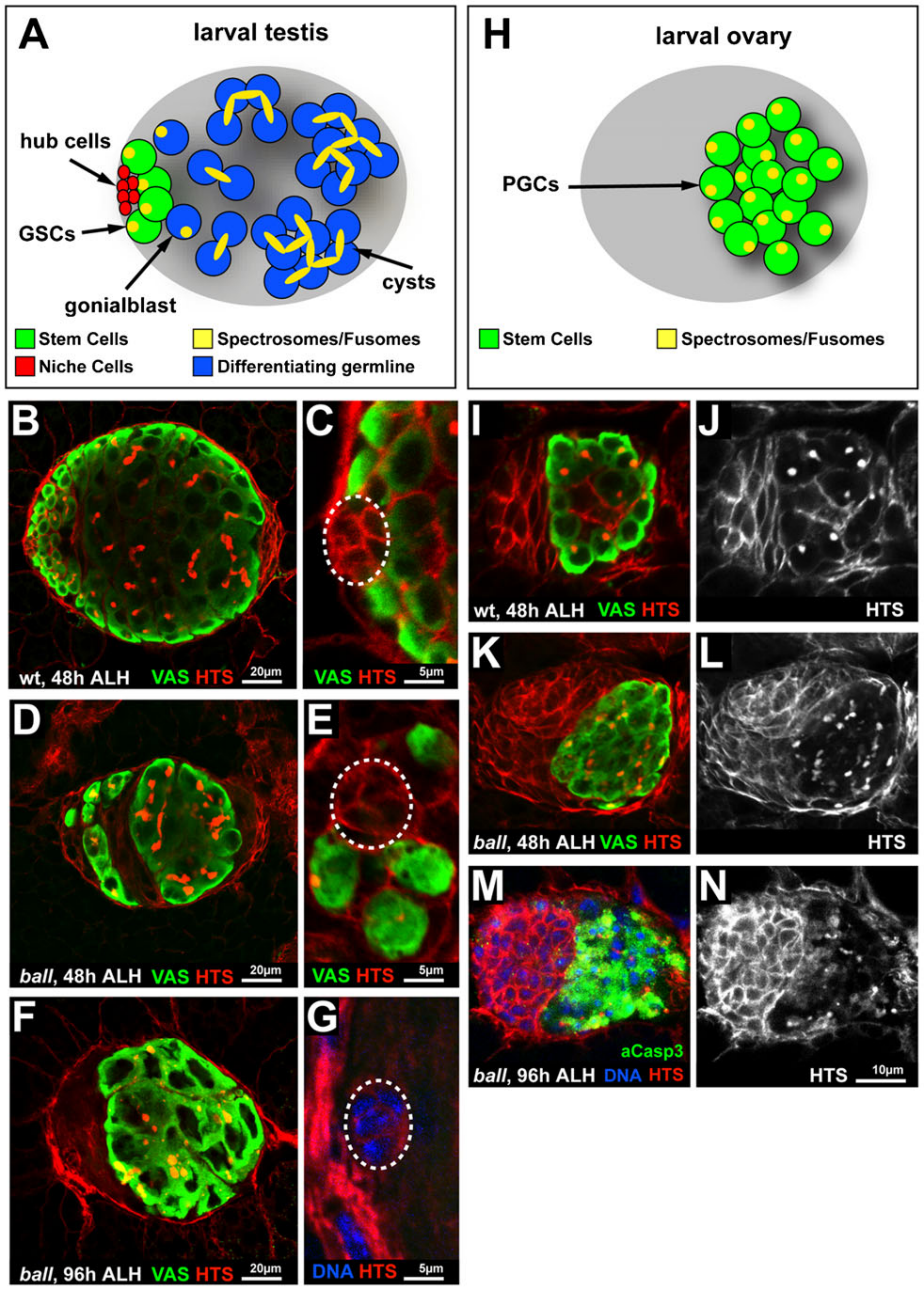
Larval gonad development requires BALL. (A) Schematic drawing of a larval testis at mid larval development that illustrates the positions of the stem cell niche formed by the hub cells (red), the GSCs (green) and the differentiating germline cells (blue). Spectrosomes in GSCs and gonialblasts as well as fusomes of cysts are illustrated in yellow. (B,C) Wild type testis at mid larval development (48 h ALH) stained for the germline marker, Vasa (VAS), and the spectrosome/fusome marker, HTS. HTS also marks the circumference of somatic cells in the testis. The enlargement (C) shows GSCs that contact the stem cell niche formed by the hub cells (dashed circle). (D,E) *ball^2^* mutant testis contain fewer germline cells than wild type at mid larval development (VAS), but fusomes of differentiating cysts are visible (HTS). The number of GSCs that contact the stem cell niche (dashed circle, E) is reduced in *ball^2^* mutant testis. (F,G) *ball^2^* mutant testis at late larval stage (96 h ALH) still contain differentiated cysts (VAS) but GSCs are absent and no longer detectable at the stem cell niche (G), which is counterstained for DNA to visualise the hub cells (dashed circle). (H) Schematic drawing of a larval ovary at mid larval development illustrating the positions of the PGCs (green) in the organ and the spectrosomes (yellow) typical at this stage. (I,J) Wild type ovary at mid larval development (48 h ALH) stained for the germline marker, Vasa (VAS), and the spectrosome/fusome marker, HTS, that also marks the circumference of somatic cells in the ovary. The HTS staining is shown separately (J) to better visualise the spectrosomes of PGCs. (K,L) *ball^2^* mutant ovary at 48 h ALH still contains viable germline cells (VAS). The HTS staining (L) revealed branched fusome-like structures instead of punctuate spectrosomes that are observed in wild type PGCs. (M,N) *ball^2^* mutant ovary at late larval stage (96 h ALH) stained for the apoptosis marker activated Caspase 3 (aCasp3), HTS and DNA. HTS staining, that marks also the circumference of somatic cells, is shown separately (N) to illustrate that cell death is largely restricted to germline cells. Anterior is to the left in the micrographs. Scale bars: 20 µm (B,D,F), 5 µm (C,E,G), 10 µm (I–N).

We next asked when the size reduction of the male gonads occurs during larval development. In early larval testes, about 8–12 PGCs adopt GSC fate after their recruitment to the somatic hub cells (Fig. 1A). GSCs then divide and give rise to self-renewed GSCs and gonialblasts, respectively. The gonialblasts undergo four incomplete cell divisions and form a 16-cell germline cyst. GSCs and differentiating cysts can be distinguished by their position in the developing testis and by the expression of the adducin-related protein, HTS (Fig. 1A). During mid larval development of wild type testis (48 h after larval hatching, ALH), HTS localises to a spectrosome in GSCs and a branched fusome in cysts, respectively (Fig. 1B,C). In *ball^2^* mutant testes 48 h ALH, differentiating germline cysts were formed, but the number of GSCs was reduced (Fig. 1D,E). By the end of larval development (96 h ALH), GSCs were completely depleted from *ball^2^* mutant testes (*n* = 21 testes). The depletion of GSCs in mutant testes was not caused by apoptosis as shown by staining for activated Caspase 3 (supplementary material Fig. S2), suggesting that *ball* is required for the maintenance of GSCs. At late larval development, *ball* mutant testes contained cysts at progressive stages of proliferation, including mature 16 cell cysts as in the case of wild type larval testes. Based on these observations, we cannot conclude that all the *ball* mutant cysts would eventually complete their differentiation program and give rise to 16 cell cysts. However, the presence of germline cysts in *ball^2^* mutant testes, which are formed by the proliferation of gonialblasts indicates that BALL is not essential for cell cycle progression and suggests that its primary function is to maintain the self-renewing capability of GSCs.

We next asked whether the absence of BALL has the same effect in the female germline. Larval ovary development depends on symmetric divisions of PGCs (Fig. 1H) (Dansereau and Lasko, 2008). Wild type ovaries during midstage of larval development contain PGCs with spectrosomes, but no differentiating cysts with HTS stained fusomes (Fig. 1I,J). In *ball^2^* mutant ovaries, however, we found fused structures in VASA expressing cells, which resembled fusomes of germline cysts (Fig. 1K,L). This result suggests that *ball* mutant PGCs differentiated prematurely. *ball^2^* mutant ovaries also contained fewer PGCs (26.7±3.6, *n* = 20 ovaries) than wild type ovaries (35.2±6.6, *n* = 23 ovaries; *P* = 6*10^−6^). The reduced number of PGCs does not result from apoptosis as staining for activated Caspase 3 showed that these differentiating germline cells were not apoptotic at mid larval development (supplementary material Fig. S2). Therefore, as observed with testes, *ball^2^* mutant germline cells in ovaries were able to proliferate. However, they proliferated at a slightly reduced rate compared to wild type. This reduction could either be due to a direct function of BALL in modulating cell cycle progression or reflect a difference in the cell cycle length between symmetrically dividing wild type PGCs and prematurely differentiating *ball^2^* mutant germline cells. At later stages of larval development, massive apoptosis in *ball* mutant PGCs was observed (Fig. 1M,N). This observation is reminiscent of previous studies on the function of *nanos* and *pumilio*, showing that mutant PGCs enter apoptosis after their premature differentiation (Gilboa and Lehmann, 2004; Wang and Lin, 2004).

### BALL is essential for self-renewal of female GSCs

**Fig. 2.**
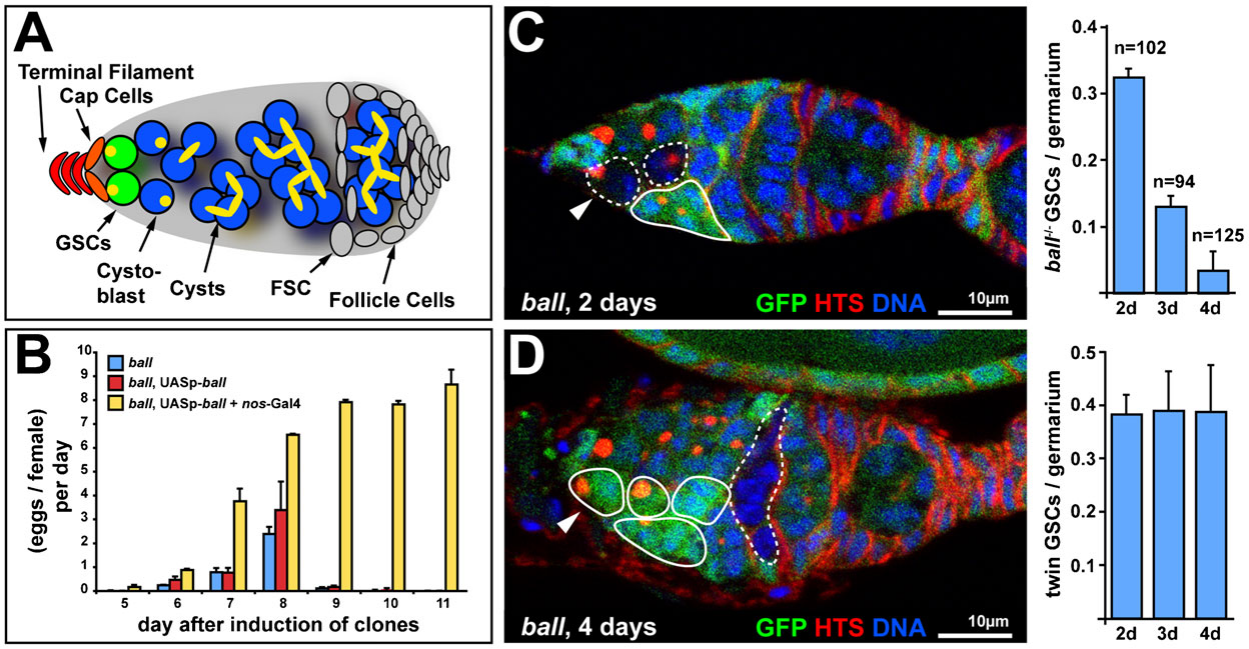
Self renewal of female GSCs depends on BALL. (A) Schematic drawing of the germarium of an adult ovary that illustrates the positions of the stem cell niche formed by the terminal filament cells (orange) and cap cells (red), the GSCs (green) and the differentiating germline cells (blue). Also indicated are follicle stem cells (FSCs) and follicle cells (grey). Spectrosomes in GSCs and cystoblasts as well as fusomes of cysts are illustrated in yellow. (B) Deposition of *ball^2^* mutant eggs was scored per indicated day and per female after induction of germline clones. Egg deposition was not sustained beyond day nine. Egg deposition continued if *ball* was expressed from a germline specific transgene (*ball*^−/−^, *UASp-ball* + *nos-Gal4*) but not by the transgene without specific expression (*ball*^−/−^, *UASp-ball*). (C) Germarium at two days AHT with *ball^2^* mutant cells (dashed circles, GFP absent) and twin clone cells (solid circles, elevated GFP) counterstained for HTS and DNA. The arrowhead marks a *ball^2^* mutant GSC, which lies next to a *ball^2^* mutant cystoblast. The graph shows averaged numbers of *ball^2^* mutant GSCs per germarium from three independent time course experiments. (D) Germarium at four days AHT with a *ball^2^* germline cyst (dashed circles, GFP absent) and a twin clone GSC (arrowhead) that generated multiple differentiating cells (solid circles, elevated GFP). The graph shows quantification of twin clone GSCs from the same set of germaria analysed in panel C. Anterior is to the left in the micrographs. Scale bars: 10 µm. The total number of germaria is given by n.

Our results suggest that the primary function of BALL is to maintain the undifferentiated state of male and female germline progenitor cells. To discriminate between a cell autonomous function of BALL in stem cells and a systemic non-autonomous function of BALL, we generated genetic mosaics in adult ovaries. GSCs and early germline cysts of adult ovaries are located at the anterior tip of the ovariole within a stem cell niche formed by somatic cap cells and terminal filament cells (Fig. 2A). In wild type, GSC divisions typically result in a self-renewed GSC and a cystoblast (CB). The CB divides four times into a 16-cell germline cyst in which cells remain interconnected due to incomplete cytokinesis. The most posterior cell in the cyst adopts an oocyte fate, whereas the remaining 15 cells differentiate into nurse cells. GSCs and CBs both carry a spherical spectrosome, whereas differentiating cysts contain a branched fusome. GSCs remain in direct contact with the stem cell niche with their spectrosome always oriented towards the niche and thus, they can be unambiguously distinguished from CBs (Fig. 2A). In order to generate genetic mosaics, we used the FLP/FRT system (Xu and Rubin, 1993) to functionally identify *ball^2^* mutant cell clones that derive from GSCs and CBs by the absence of an *ovo^D1^* expressing transgene. *ovo^D1^* expression induces apoptosis in germline cysts (Perrimon, 1998). Thus, only cysts that derive from a *ball^2^* mutant GSC or CB lack the *ovo^D1^* expressing transgene and hence, can develop into eggs. We generated *ball^2^* mutant germline clones by heatshock-induced *flipase* expression (Xu and Rubin, 1993) and monitored the number of eggs deposited at various time points after heatshock treatment (AHT).

The results show that *ball^2^* mutant eggs were first deposited six days AHT (Fig. 2B). This indicates that *ball^2^* mutant germline clones can differentiate into mature eggs. However, *ball^2^* mutant egg deposition vanished after three days (Fig. 2B). Germline specific expression of *ball*, achieved by combining a *UASp*-*ball*-*EGFP* transgene with a *nos*-*GAL4* driver transgene, restored the capacity of *ball^2^* mutant GSCs to sustain continuous egg production (Fig. 2B). In conclusion, these results show that germline autonomous expression of *ball* is sufficient to allow continuous egg production and, therefore, also to maintain self-renewing GSCs.

The lack of continuous egg production from *ball* mutant germline clones could be explained by a defect in germline differentiation which, however, was masked by the perdurance of BALL protein after clone induction. In order to directly address the function of *ball* for GSC self-renewal and germline differentiation, we generated genetic mosaics identifying *ball^2^* mutant cell clones by the absence of GFP expression (Fig. 2C). In three replicate time course experiments, we found 0.32±0.017 *ball^2^* mutant GSCs per germarium (*n* = 102) at two days AHT. This number decreased to 0.13±0.021 (*n* = 94 germaria) and 0.034±0.039 (*n* = 125 germaria) three and four days AHT, respectively. To distinguish the loss of *ball^2^* mutant GSCs from the regular turnover of GSCs, we counted the number of wild type ‘twin clone’ GSCs in the same set of germaria. These GSCs are initially generated at the same frequency as mutant GSCs in genetic mosaics and can be identified by their increased GFP fluorescence intensity (Fig. 2D). The number of twin clone GSCs remained constant over the time course (0.38±0.049, 0.39±0.11 and 0.39±0.11 after two, three and four days, respectively), showing that *ball^2^* mutant GSCs were not lost due to regular turnover. Furthermore, *ball^2^* mutant cells did not undergo cell death during the time period as shown by staining for activated Caspase 3 (supplementary material Fig. S3). These results show that *ball^2^* mutant GSCs had lost their capacity to self-renew and left the niche.

**Fig. 3.**
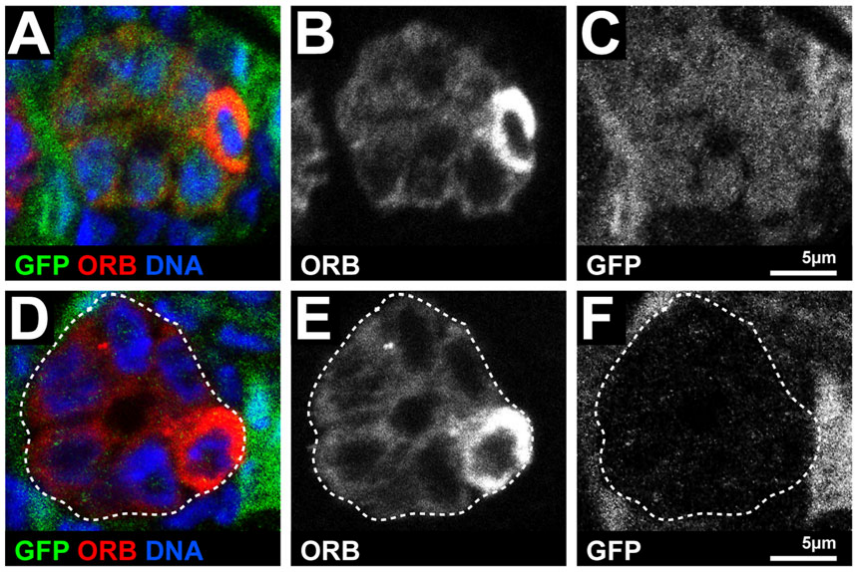
ORB localisation is not affected in *ball^2^* mutant egg chambers. (A–C) In wild type egg chambers (GFP positive), ORB is expressed in all germline cells and the protein is enriched in the oocyte located at the posterior of the egg chamber. Staining for ORB and detection of GFP are shown in separate panels for clarity, cells were counterstained for DNA. (D–F) *ball^2^* mutant egg chambers are identified by the absence of GFP detection. ORB distribution is indistinguishable from wild type, indicating normal specification of the oocyte. Cells were counterstained for DNA. Anterior is to the left in the micrographs. Scale bars: 5 µm.

To analyse the differentiation of *ball^2^* mutant germline clones, we stained for the ORB protein, which becomes progressively enriched in the oocyte during cyst differentiation (Christerson and McKearin, 1994) (Fig. 3A–C). The distribution of ORB was similar in *ball^2^* mutant and wild type cysts (Fig. 3D–F), indicating that oocyte specification was normal. However, we noted that in about half of the *ball^2^* mutant germline cysts a single nurse cell degenerated and that all oocytes displayed the characteristic defect in chromatin organisation that has been previously reported (Ivanovska et al., 2005) (supplementary material Fig. S4). Nevertheless, the overall differentiation of *ball* mutant cysts continued normally beyond the mitotic stages (supplementary material Fig. S4). This finding is consistent with the formation of mature eggs that were observed with the germline clone experiments (see above).

Using anti-BALL antibodies, we did not detect residual BALL protein in differentiating *ball* mutant germline cysts (data not shown). Germline cyst formation requires four cell divisions during which residual BALL protein present in a *ball* mutant CB would be diluted. Residual BALL protein in GSCs would similarly be diluted by cell divisions, i.e. *ball* mutant GSCs were lost within 4 days, which allows about four divisions of GSCs. Although we can formally not rule out residual BALL protein below the detection limit in differentiating cysts, the data strongly support that the requirement of BALL for GSC self-renewal is at least by far more critical than for germline differentiation.

### BALL is essential for self-renewal of male GSCs

**Fig. 4.**
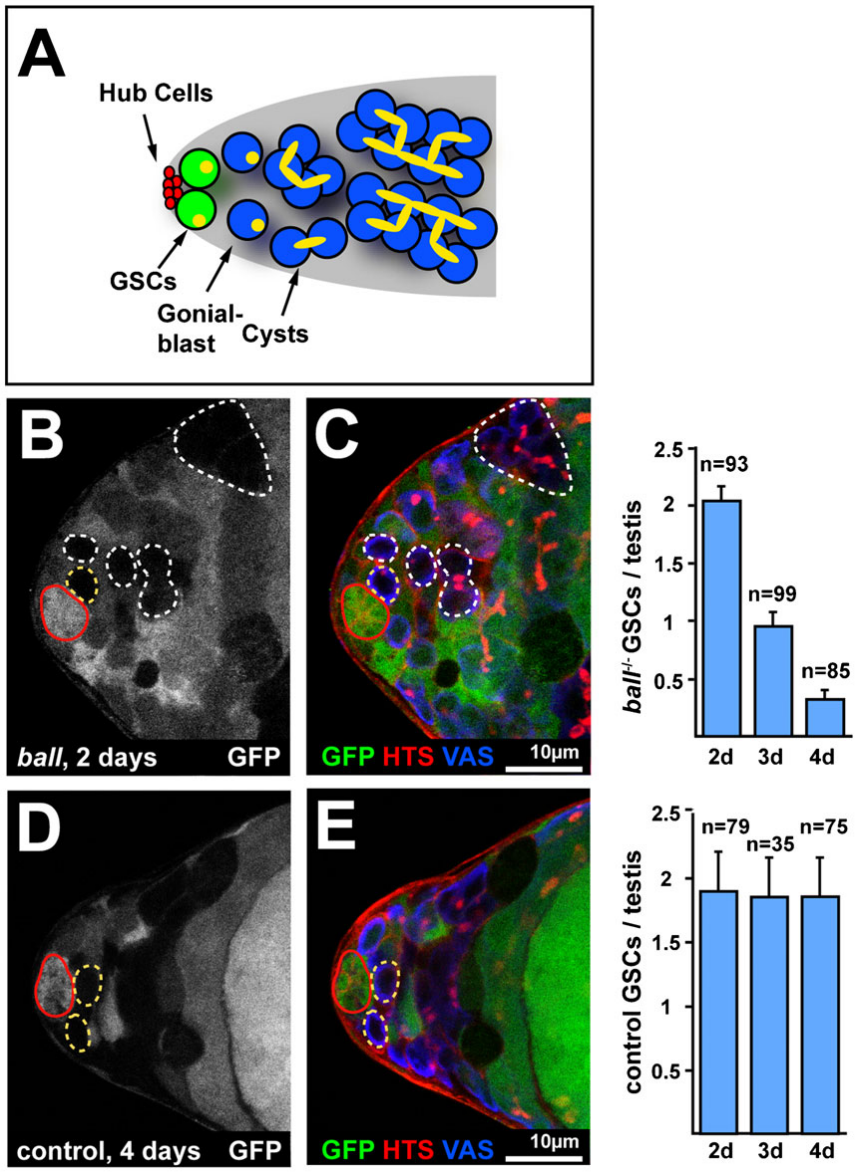
Self-renewal of male GSCs depends on BALL. (A) Schematic drawing of an adult testis tip that illustrates the positions of the stem cell niche formed by the hub cells (red), the GSCs (green) and the differentiating germline cells (blue). Spectrosomes in GSCs and gonialblasts as well as fusomes of cysts are illustrated in yellow. (B,C) Testis tip at two days AHT with *ball^2^* mutant cells (dashed circles, GFP absent) counterstained for HTS and the germline marker Vasa (VAS). The GFP detection channel is shown separately. *ball^2^* mutant GSCs (yellow dashed) are identified based on their direct contact to the hub cells (red circle, VAS absent). The graph shows average numbers of *ball^2^* mutant GSCs per testis from three independent time course experiments. (D,E) Testis tip at four days AHT with wild type control GSCs that are marked by the absence of GFP and detected as described in panels B and C. The graph shows average numbers of control GSCs per testis from three independent time course experiments. Anterior is to the left in the micrographs. Scale bars: 10 µm. The total number of testes is given by n.

To assess whether the function of BALL for self-renewal of GSC is specific for the female germline, we examined the function of BALL in GSCs of adult testes. In adult testes, about nine GSCs are in direct contact with the stem cell niche formed by hub cells at their anterior tips (Fig. 4A). Both, GSCs and the differentiating daughter cells, called gonialblasts, carry a spectrosome with a random orientation. GSCs can be unambiguously distinguished from gonialblasts by their position adjacent to the niche. We generated genetic mosaics with *ball^2^* mutant cells visualised by the absence of GFP expression (Fig. 4B,C). Instead of a twin-clone analysis, we generated GFP marked wild type cells, since twin clone analysis was not reliable in testes GSCs (Fig. 4D,E). We counted the number of marked GSCs per testis, showing that two days AHT, both *ball^2^* mutant GSCs and wild type GSCs were equally frequent, with 2.04±0.15 (*n* = 93 testes) and 1.9±0.42 (*n* = 79 testes) GSCs per testis, respectively. After three and four days AHT, however, the frequency of *ball^2^* mutant GSCs decreased to 0.95±0.17 (*n* = 99 testes) and 0.32±0.11 (*n* = 85 testes), respectively, whereas the wild type control GSCs were maintained at the originally observed high frequency, i.e. 1.85±0.4 (*n* = 35 testes) and 1.86±0.56 (*n* = 75 testes) GSCs, respectively. No cell death was found in *ball^2^* mutant GSCs between day two and four AHT (*n*≥50 testes per day). These results establish that BALL has a critical function in maintaining self-renewing GSCs both in the male and female germline.

### BALL is essential for functional somatic FSCs in the ovary

We next asked whether the function of BALL is limited to stem cells of the germline and thus, we extended our analysis on the somatic cell lineage in ovaries. During egg development, germline cysts become encapsulated by epithelial follicle cells (FCs), which are continuously generated by somatic follicle stem cells (FSCs) in the germarium (Fig. 2A).

**Fig. 5.**
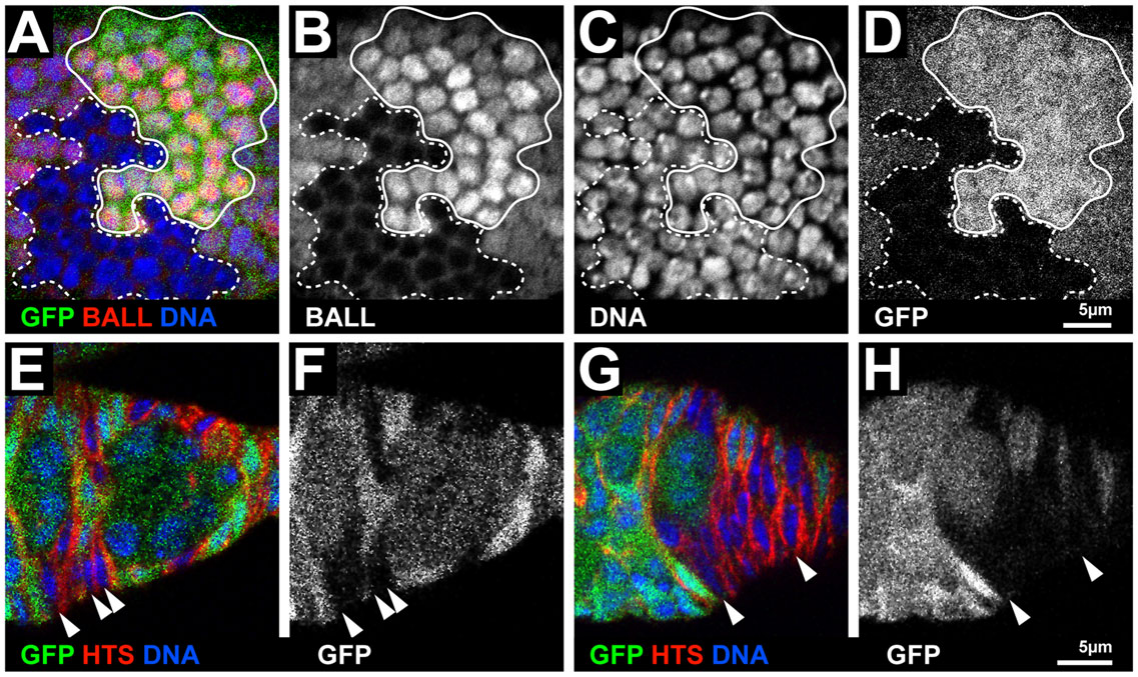
Proliferation of *ball^2^* mutant follicle stem cells and follicle cells. (A,B) Two days after induction of genetic mosaics, germaria still contained early follicle cells that were mutant for *ball^2^* (arrowheads, absent GFP signal). This indicates the presence of functional *ball^2^* mutant FSCs. Germaria were counterstained stained for DNA and for HTS, which marks the circumferences of somatic cells. (C,D) Ten days after induction of genetic mosaics that contained wild type control GFP negative cells, we detected functional FSCs by the presence of large GFP negative cell clones of early follicle cells (in between arrowheads). We did no longer find *ball^2^* mutant early follicle cells ten days after induction of respective genetic mosaics, indicating that *ball^2^* mutant FSCs were not maintained. (E–H) Two days after induction of *ball^2^* mutant genetic mosaics we stained differentiating follicle cells that surround egg chambers for BALL and for HTS. Cell clones in these cells do not derive from recombinant FSCs but from a recombination event in a proliferating follicle cell. Therefore we found *ball^2^* mutant cell clones (GFP absent, dashed circle) associated with wild type twin clones (GFP increased, solid circle). Cell numbers in *ball^2^* mutant clones were indistinguishable from cell numbers in twin clones, indicating that *ball^2^* mutant follicle cells proliferate at wild type rate. Anterior is to the left in the micrographs. Scale bars: 5 µm.

Before FCs terminally differentiate, they undergo multiple cell divisions in the follicle epithelium around the egg chambers. To assess the proliferation of wild type and *ball* mutant FCs, we compared the sizes of corresponding mutant and wild type twin clones (Fig. 5A–D). The average ratio of cell numbers in *ball^2^ versus* wild type twin clone pairs was 1.06±0.45 (*n* = 31 pairs), indicating that wild type and *ball^2^* mutant FCs proliferated at a similar rate.

Since FCs are continuously displaced from the germarium together with the developing egg chambers, the functional availability of FSCs can be monitored by the appearance of newly generated FCs in the germarium. Two days after induction of clones, 45% of the germaria (*n* = 33) contained *ball^2^* mutant FCs (Fig. 5E,F). However, ten days after induction of clones, no *ball* mutant FCs were detected (*n* = 35), but 40% of the germaria (*n* = 30) contained wild type twin clone FCs and non-mutant control clones were also still present (Fig. 5G,H). Because we did not directly identify FSCs as we did in the case of the GSCs, we could not discriminate whether *ball* mutant FSCs died, stopped proliferation or have been dislocated from the niche and differentiated. The *ball* loss of function phenotype in the somatic lineage of the ovary however shares extensive similarity with the germline phenotype in a sense that BALL is dispensable for the proliferation of differentiating cells but required for the continuous supply of cells that maintains tissue homeostasis through self-renewing stem cells.

### BALL is not required for BMP signalling in female GSCs

**Fig. 6.**
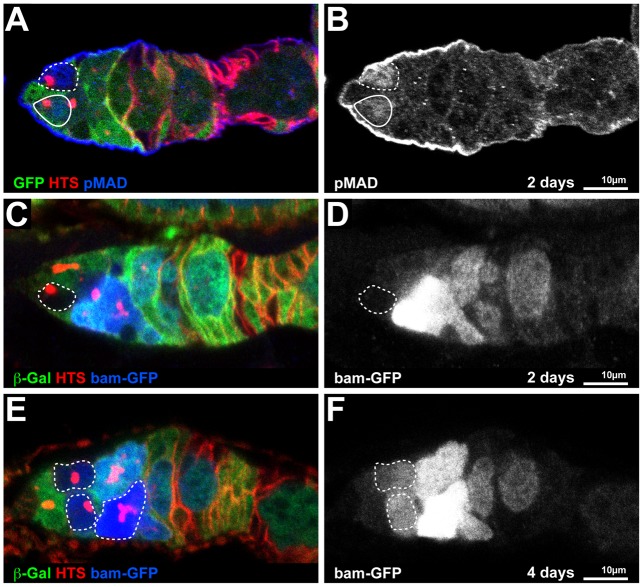
BMP signalling in *ball^2^* mutant germline cells. (A,B) Germarium at two days AHT with a *ball^2^* mutant GSC (dashed circle, GFP absent) and a wild type GSC (solid circle) counterstained for HTS and phosphorylated MAD protein (pMAD). The pMAD staining is shown separately to illustrate that loss of BALL does not interfere with MAD phosphorylation. (C,D) Germarium at two days AHT with a *ball^2^* mutant GSC (dashed circle) marked by the absence of β-Galactosidase (β-Gal) expression and counterstained for HTS. Expression of *bam-GFP*, which is not detectable in the *ball^2^* mutant GSC, is shown as a separate channel. (E,F) Germarium at four days AHT with multiple, differentiating *ball^2^* mutant germline cells (dashed circle, absent β-Gal) counterstained for HTS. Expression of *bam-GFP* is upregulated in the differentiating *ball^2^* mutant germline cysts. Anterior is to the left in the micrographs. Scale bars: 10 µm.

The stem cell populations in both male and female gonads rely directly or indirectly on BMP signalling. We used the female GSC system, in which the requirement of BMP signalling is most prominent, to address the relevance of BALL to BMP signalling. We assayed two hallmarks of BMP signalling in GSCs by immunostainings, i.e. the phosphorylation of the transcription factor MAD (pMAD) and the repression of the *bag of marbles* (*bam*) gene (Song et al., 2004). In wild type as well as in *ball^2^* mutant GSCs, pMAD was stained in GSCs (Fig. 6A,B). Consistent with functional BMP signalling and MAD phosphorylation, *bam* was properly repressed in *ball^2^* mutant GSCs (Fig. 6C,D) and *bam* expression was normally upregulated in differentiating cells of *ball^2^* mutant cysts (Fig. 6E,F). In conclusion, these observations indicate that BALL acts independently or downstream of the BMP signalling pathway in female GSCs.

### Loss of *ball* mutant GSCs from the niche does not dependent on their differentiation

**Fig. 7.**
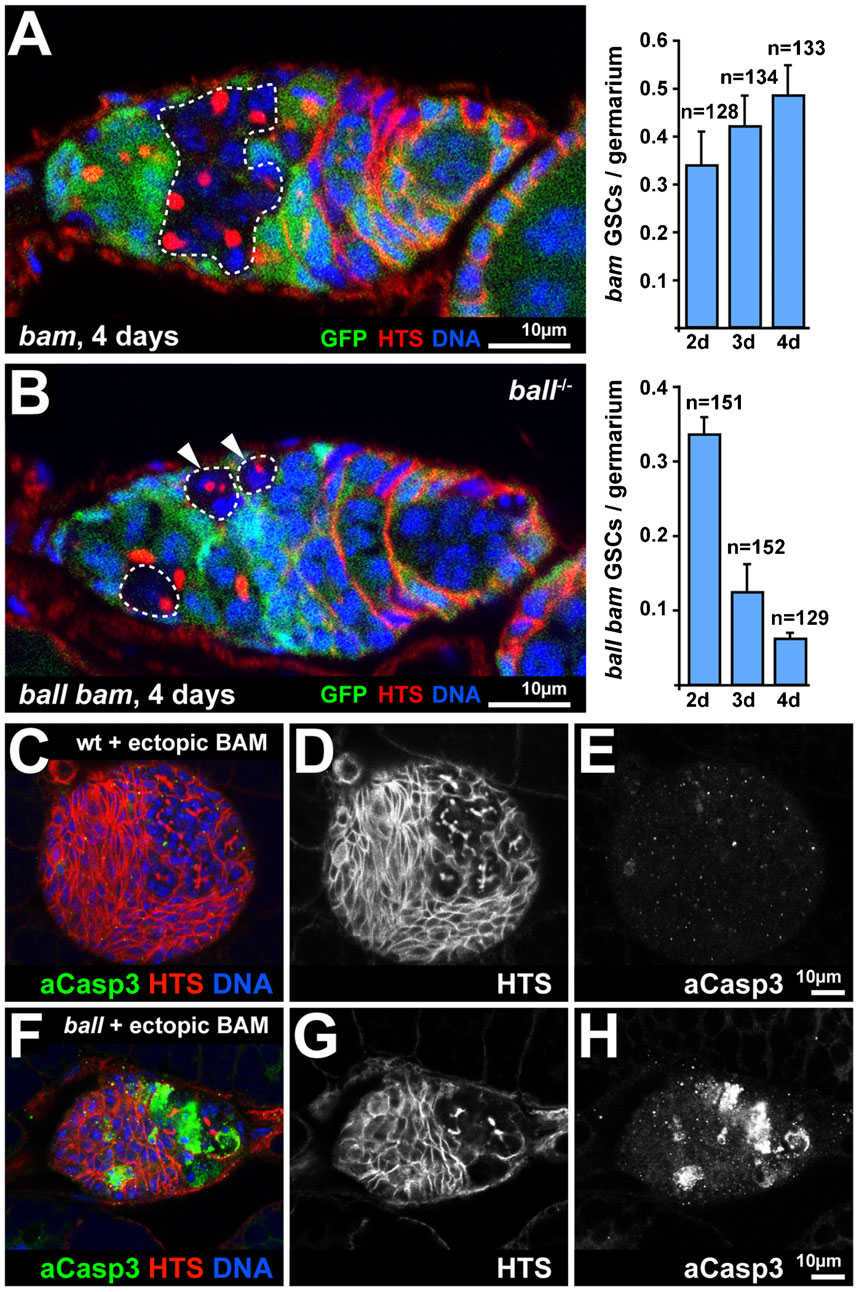
BALL is required for GSC competitiveness and for survival of differentiation blocked germline cells. (A) Germarium at four days AHT with *bam^Δ86^* mutant germline cells (dashed circle, GFP absent) counterstained for HTS and DNA. *bam^Δ86^* mutant cells divide outside the stem cell niche and maintain stem cell like spectrosomes as visualised by HTS. The graph shows average numbers of *bam^Δ86^* mutant GSCs that are in direct contact with the stem cell niche per germarium from three independent time course experiments. (B) Germarium at four days AHT with *ball^2^ bam^Δ86^* double mutant germline cells (dashed circle, GFP absent) counterstained for HTS and DNA. *ball^2^ bam^Δ86^* double mutant germline cells do not form tumours and eventually degenerate outside the stem cell niche (arrowheads). The graph shows average numbers of *ball^2^ bam^Δ86^* double mutant GSCs per germarium from three independent time course experiments, which reveals stem cell loss similar to *ball^2^* single mutants (compare to Fig. 2C). (C–E) Ovary at late larval stage (96 h ALH) stained for the apoptosis marker activated Caspase 3 (aCasp3), HTS and DNA. Heat induced expression of *bam* in this ovary led to the differentiation of PGCs. The HTS detection channel is shown separately (D) to better visualise fusomes of differentiating germline cysts and the activated Caspase 3 detection channel (E) is shown to demonstrate that the differentiation of PGCs did not result in apoptosis. (F–H) *ball^2^* mutant ovary treated and stained analogous to panels C–E. Heat induced expression of *bam* in this mutant ovary did not fully suppress the apoptosis of *ball^2^* mutant germline cells (H) that was observed without *bam* overexpression (compare to Fig. 1M,N). Anterior is to the left in the micrographs. Scale bars: 10 µm. The total number of germaria is given by n.

Upregulation of the differentiation factor BAM is critically required for the differentiation of CBs into germline cysts (Chen and McKearin, 2003; Song et al., 2004). *bam^Δ86^* single mutant GSCs generate daughter cells that fail to differentiate but instead develop stem cell-like tumours in the anterior region of the germarium (Fig. 7A). We generated genetic mosaics with *ball^2^ bam^Δ86^* double mutant GSCs and asked whether their differentiation into cysts is a prerequisite for the elimination of *ball* mutant GSCs from the niche. *ball^2^ bam^Δ86^* double mutant GSCs, were lost from the stem cell niche (Fig. 7B) as described for *ball^2^* single mutant GSCs (Fig. 2C). Moreover, in a time course experiment, we could not detect apoptotic *ball^2^ bam^Δ86^* double mutant GSCs in the niche (supplementary material Fig. S3D). Importantly, niche-detached *ball^2^ bam^Δ86^* double mutant cells did not differentiate into germline cysts (Fig. 7B). Thus, loss of BALL does not bypass the requirement of BAM for differentiation. These results indicate that *ball* mutant GSCs are not eliminated from the niche because they ectopically initiate differentiation, but through a differentiation-independent process.

In addition to producing differentiation defective CBs, *bam^Δ86^* mutant GSCs become “super-competitive stem cells”, i.e. they displace wild type GSCs from the stem cell niche (Fig. 7A) through cellular competition (Jin et al., 2008; Rhiner et al., 2009). The fact that this increased competiveness of *bam^Δ86^* mutant GSCs is abrogated by the loss of *ball* in *ball^2^ bam^Δ86^* double mutant GSCs suggests that BALL is a factor that mediates cellular competition and that *ball* mutant GSCs are lost from the stem cell niche due to reduced competitiveness and independent of their ability to differentiate.

We noticed that *ball^2^ bam^Δ86^* double mutant cells did not form stem cell tumours after leaving the niche but eventually degenerated (Fig. 7B). This finding is different from *ball^2^ bam^Δ86^* double mutant GSCs that were not degenerating while they resided in the niche (supplementary material Fig. S3D), i.e. in an environment that promotes the undifferentiated state of GSCs. Similarly to *ball^2^ bam^Δ86^* double mutant germline cells outside the niche, larval PGCs lack BAM expression and *ball^2^* mutant PGCs undergo apoptosis (Fig. 1M,N). Therefore, we asked whether there is a general synergistic requirement of BAM and BALL for the survival of differentiating germline cells. Ectopic expression of *bam* from a transgene was sufficient to induce differentiation of wild type PGCs (Fig. 7C–E), but did not maintain the survival of differentiating *ball^2^* mutant PGCs (Fig. 7D–F). These observations argue against an interdependent requirement of BAM and BALL for the survival of differentiating germline cells.

### BALL activity maintains the stem cell specific organization of the nucleolus

**Fig. 8.**
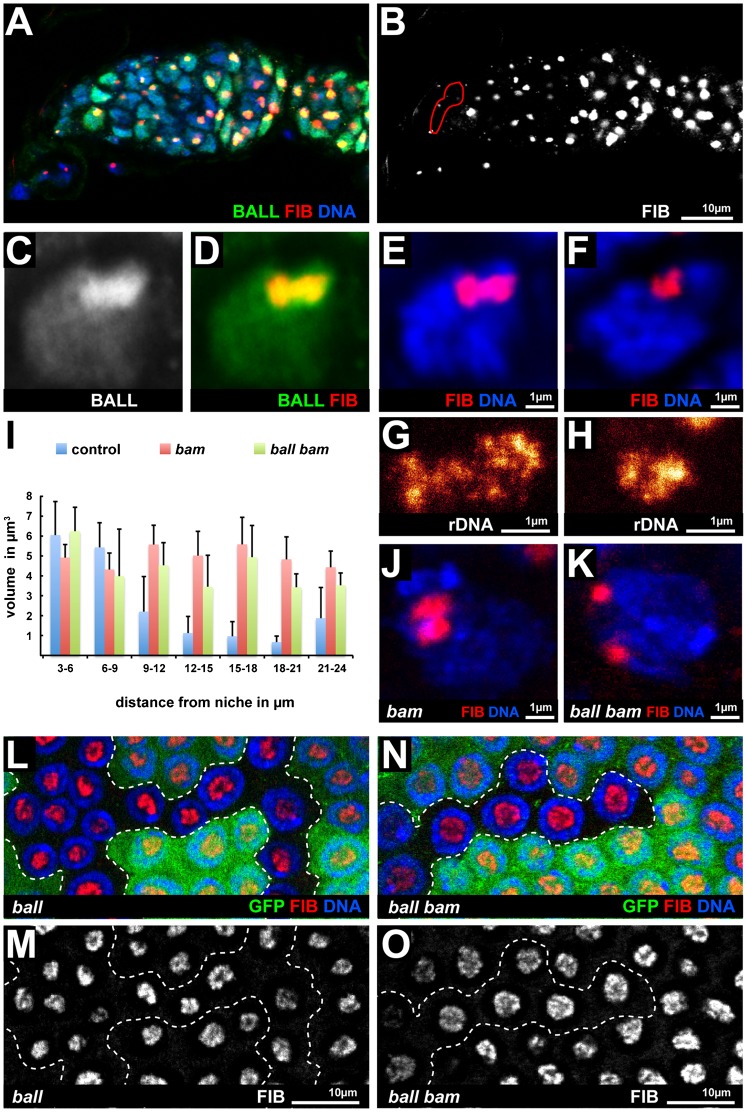
BALL is required for maintenance of the enlarged nucleolus in GSCs. (A,B) Wild type germarium stained for BALL, Fibrillarin (FIB) and DNA. The position of the stem cell niche is indicated by a red circle in panel B. (C–E) Enlargement of a GSC from the same staining showing that BALL is enriched in the nucleolus, which is identified by Fibrillarin staining. (F) In germline cyst cells the size of the nucleolus is decreased based on Fibrillarin staining. (G,H) High resolution imaging of DNA-FISH against ribosomal genes (rDNA) reveals that the decrease in nucleolar size observed by Fibrillarin staining is paralleled by a compaction of rDNA repeats from GSCs (G) to differentiating cyst cells (H). (I) Plot showing the size of the nucleolus by the volume of Fibrillarin staining in µm^3^ as a function of the distance from the stem cell niche. The data are derived from genetic mosaics that either contained GFP negative wild type cells (control, *n* = 134 cells), *bam^Δ86^* mutant cells (*bam*, *n* = 84 cells) or *ball^2^ bam^Δ86^* double mutant cells (*ball bam*, *n* = 110 cells). Nucleolar size decreases in wild type cells that differentiate and move away from the stem cell niche (blue bars). *bam^Δ86^* cells do not differentiate but proliferate at GSC-like cells outside the niche and maintain a stem cell like nucleolar size (red bars). *ball^2^ bam^Δ86^* double mutant cells with an intact nucleolus show a slightly decreased size compared to *bam^Δ86^* cells but an enlarged nucleolus compared to differentiating control cells. (J) The nucleolus of *bam^Δ86^* cells resembled the nucleolus of wild type GSCs in shape and size. (K) In about half of the *ball^2^ bam^Δ86^* double mutant cells we found a fragmented or degenerated nucleolus based on Fibrillarin staining. (L–O) Late stage endoreduplicating follicle cells, which massively increases the size of their nucleoli, stained for DNA and Fibrillarin (FIB). This size increase was neither affected in *ball^2^* mutant cell clones (dashed lines in L,M) nor in *ball^2^ bam^Δ86^* double mutant cell clones (dashed lines in N,O), which were identified by an absent GFP signal. Anterior is to the left in the micrographs (A,B). Scale bars: 10 µm (A,B), 1 µm (C–H,J,K), 10 µm (L–O).

Loss of BALL-dependent phosphorylation of BAF was identified as the primary cause for the chromatin organization defects in *ball* mutant oocytes (Lancaster et al., 2007). However, we did not observe severe chromatin organization defects in *ball^2^* mutant GSCs or cells other than the oocyte (supplementary material Fig. S5). This observation suggests that BALL is not generally involved in global chromatin organization, but might affect specific aspects of it in specific cells or organs. While reinvestigating the subcellular localization of BALL, we found that BALL not only covers the entire chromatin (Aihara et al., 2004), but is also highly enriched in the nucleolus (Fig. 8A–E). The nucleolus is assembled around the chromatin region that harbours the rDNA repeats and can be visualized with antibodies against Fibrillarin that marks the granular zone of the nucleolus. Compared to GSCs, the size of the nucleolus becomes significantly decreased during the differentiation of germline cysts, which suggests a reorganization of the nucleolar content, and it increases again when postmitotic germline cyst cells start to polyploidize (Neumüller et al., 2008). To see whether this change of nucleolar organization includes a reorganization of the chromatin that carries the rDNA genes, we performed DNA-FISH experiments in combination with high-resolution STED microscopy. Comparing GSCs to differentiating germline cells revealed that the rDNA genes in the nucleolus become significantly compacted during differentiation (Fig. 8G,H).

The volume of nucleoli, as assayed by Fibrillarin staining, continuously decreased with the distance of germline cells from the stem cell niche, which reflects the differentiation process of wild type germline cysts (Fig. 8I). *bam^Δ86^* mutant tumourous germline cells maintained a GSC-like nucleolar size irrespective of the distance to the stem cell niche (Fig. 8I,J), consistent with the fact that these cells proliferate like GSCs outside the niche. Hence, we used the *bam^Δ86^* mutant cells to address whether BALL is required for a GSC-like organization of the nucleolus. In *ball^2^ bam^Δ86^* double mutant mosaics, the nucleoli of about half of the mutant cells were only slightly smaller (53%, *n* = 110 cells) than the nucleoli of *bam^Δ86^* mutant cells (Fig. 8I). This observation suggests that the mechanisms that induce the expansion of nucleoli in GSCs are still functional in the absence of BALL. Maintenance of this nucleolar expansion is however dependent on BALL activity, since in the other half of the mutant cells the nucleoli were either fragmented or completely disintegrated (47%, *n* = 110 cells) (Fig. 8K). Interestingly, we did not observe degeneration of nucleoli in those *ball^2^ bam^Δ86^* double mutant cells that resided in the stem cell niche where self-renewal is supported by BMP signalling. This result suggested that BALL is either directly or indirectly required to maintain the nucleolus of GSC-like tumorous cells that cannot differentiate due to the absence of BAM, but no longer receive the niche derived maintenance signals. To see whether BALL is required for maintenance of expanded nucleoli in cells that normally differentiate, we asked whether the nucleoli of follicle cells, which increase their nucleoli during endoreduplication, are affected by the absence of BALL. Staining of these nucleoli with Fibrillarin antibodies showed that the nucleoli of neither *ball^2^* mutant (Fig. 8L,M) nor *ball^2^ bam^Δ86^* double mutant cells were affected in differentiating follicle cells (Fig. 8N,O). These observations suggest that BALL is not generally required for the generation or maintenance of an expanded nucleolus but has a specific function in tumorous stem cells.

## DISCUSSION

Our study shows that the VRK-1 kinase BALL is required for self-renewal of germline stem cells in *Drosophila*, including the symmetrically amplifying PGCs of larvae and both male and female GSCs. These stem cells are actively maintained undifferentiated and they require BMP signalling for self-renewal that emanates from their cellular niche environments (Losick et al., 2011). In *ball^2^* mutant female GSCs, where the requirement of BMP signalling for self-renewal is most pronounced, known targets of BMP signalling are regulated as in wild type GSCs. This indicates that BALL participates neither in the transmission nor the regulation of BMP signalling, and that it is needed to maintain stem cell character in a cell autonomous manner, irrespective of the tissue-specific maintenance signals that emanate from the niches.

The loss of self-renewing stem cells could be caused by the induction of ectopic differentiation in these cells. We blocked the differentiation pathway in *ball* mutant female GSCs by removing also the central differentiation factor BAM. The results suggest that *ball* mutant GSCs are not eliminated from the stem cell niche because they initiate germline differentiation but that the GSCs differentiate because they lost the capacity for self-renewal.

It is unclear by which mechanism BALL mediates the ability for GSC self-renewal. In ovaries, GSCs and FSCs undergo a regular turnover and are continuously replaced in the niche either by their own daughter cells or by symmetric divisions of the neighbouring stem cells (Nystul and Spradling, 2007; Xie and Spradling, 2000). The replacement of GSCs involves competition between stem cells. Cells lacking BAM for instance, successfully displace less competitive wild type stem cells in the niche (Jin et al., 2008). However, if BALL is additionally removed from *bam* mutant cells, they appear to loose their competitive advantage. The molecular basis of stem cell competiveness is still poorly understood. However, it has been shown that overexpression of the *Drosophila* dMyc transcription factor *diminutive* enhances the competitiveness of GSCs (Rhiner et al., 2009) and causes significantly enlarged nucleoli and increased rRNA expression in epithelial cells (Grewal et al., 2005). These observations suggest a correlation between ribosome biogenesis and GSC competitiveness. Downregulation of ribosome biogenesis appears in fact to be directly required for germ cell differentiation, since BAM activates the Mei-P26 protein which downregulates the expression of dMyc. Furthermore, when overexpressed from a transgene, dMyc abrogates the tumour growth phenotype and the size increase of nucleoli in *bam* mutant cells (Neumüller et al., 2008). Additional support for the proposal that increased ribosome biogenesis in stem cells is crucial for their competitiveness and for maintaining their undifferentiated state derives from studies on *wicked*. Wicked is an essential component of the U3 snoRNP pre-rRNA processing, which is required to maintain the self-renewal of GSC (Fichelson et al., 2009). Our findings that BALL is enriched in stem cell nucleoli and required for the structural integrity of nucleoli in tumourous GSCs provide a plausible link between ribosome biogenesis and BALL-dependent competitiveness of GSCs.

Once displaced from the niche, *ball^2^* mutant female GSCs differentiate according to their germline fate with only minor defects. We did not address whether the differentiation of *ball* mutant cells is fully completed like in the respective wild type lineages in systems other than the adult female germline, but we found, irrespective of the system we looked at, that BALL is not strictly required as proliferation factor. Especially the analysis of dividing follicle cells showed that BALL is not a cell cycle regulator. With two remarkable exceptions, BALL is also not essential for cellular survival. These exceptions, i.e. *ball* mutant PGCs at late larval stages and *ball^2^ bam^Δ86^* double mutant germline cells outside the ovarian niche, represent conditions in which differentiation is either not supported by the tissue or not possible due to the lack of a differentiation factor. Therefore, it is tempting to speculate that BALL becomes only essential for cellular survival, when the *ball* mutant stem cells are unable to ‘escape’ from self-renewal into differentiation. Although *ball* clearly has multiple functions, e.g. oocyte chromatin organization or modulation of female PGC proliferation rate, the common defect observed in all systems examined so far is a failure in maintaining pools of undifferentiated cells. Since BALL is not an essential proliferation factor, our data suggest that also the additional defects in *ball* mutant larvae, i.e. lacking imaginal discs and degenerate brains, could be due to premature loss of undifferentiated progenitor or stem cells. Analysis of these systems will eventually show whether BALL is broadly required to maintain the undifferentiated state of cells during development.

## MATERIALS AND METHODS

### Fly strains

The *ru ca e ball^2^* chromosome was generated by imprecise excision of P{*EP*}*ball^EP863^* (*ball^1^*). The chromosomes P{*neoFRT*}82B, P{*neoFRT*}82B *e ball^2^*, P{*neoFRT*}82B *e bam*^Δ86^ and P{*neoFRT*}82B *e bam*^Δ86^ *ball^2^* were constructed by meiotic recombination. The transgene P{*w^+mC^ UASp-ball.T:Avic/EGFP = pballE*}2.1 was used for BALL-EGFP expression.

Clones with *ovo^D1^* transgenes were induced in 1–3 (*ball*):

(1) *y*^1^ w* P{*ry*^+^, *hs-FLP*}1/w*; P{*neoFRT*}82B P{*ovoD1-18*}3R/P{*neoFRT*}82B *e ball^2^*
(2) *y*^1^ w* P{*ry*^+^, *hs-FLP*}1/w*; P{*pballE*}2.1/P{*GAL4-nos.NGT*}40; P{*neoFRT*}82B P{*ovoD1-18*}3R/P{*neoFRT*}82B *e ball^2^*
(3) *y*^1^ w* P{*ry*^+^, *hs-FLP*}1/w*; P{*pballE*}2.1/*wg*^Sp1^; P{*neoFRT*}82B P{*ovoD1-18*}3R/P{*neoFRT*}82B *e ball^2^*

GFP-marked clones were induced in:

(4) *y*^1^ w* P{*ry*^+^, *hs-FLP*}1/w*; P{*neoFRT*}82B P{*Ubi-GFP*}83/P{*neoFRT*}82B *e ball^2^* (males: w* X-chromosome replaced for Y chromosome)
(5) *y*^1^ w* P{*ry*^+^, *hs-FLP*}1/w*; P{*neoFRT*}82B P{*Ubi-GFP*}83/P{*neoFRT*}82B *e bam*^Δ86^
(6) *y*^1^ w* P{*ry*^+^, *hs-FLP*}1/w*; P{*neoFRT*}82B P{*Ubi-GFP*}83/P{*neoFRT*}82B *e bam*^Δ86^ *ball^2^*
(7) *y*^1^ w* P{*ry*^+^, *hs-FLP*}1/*Y*; P{*neoFRT*}82B P{*Ubi-GFP*}83/P{*neoFRT*}82B
(8) *y*^1^ w* P{*ry*^+^, *hs-FLP*}1/P{*bamP-GFP*}, w*; P{*neoFRT*}82B P{*arm-lacZ.V*}83B/P{*neoFRT*}82B *e ball^2^*

Female flies were used (1) to assay the *ball* requirement in egg production, (2) for germline specific rescue and (3) to serve as control to show that rescue was not caused by basal expression of the *ball* transgene. Female and male flies (4) were used to generate marked *ball^2^* mutant clones in germaria or testis. Control non-mutant clones were induced in male flies (7). Repression of a *bam-GFP* reporter construct was assayed in (8). To generate *bam* mutant and double mutant clones, the loss of function allele *bam*^Δ86^ was used in (5) and (6), respectively.

To obtain *ball^2^* mutant larvae, this allele was balanced with *TM3*, *Ser^1^*, *P*{*ActGFP*}*JMR2* and newly hatched GFP negative larvae were collected over a two hours interval. Controls and were treated identically. Larvae were subsequently reared at a controlled density before dissection. Induced expression of *bam* through the P{*hs-bam.O*}*18d* integration was accomplished by heat shocks (45 min, 37.5°C) every 24 hours starting from 12 h after larval hatching.

### Induction of clones

Flies aged 0–2 d were fed on yeast for 1 d at 25°C. Subsequently, three heat shocks (1 h, 38°C) were applied in 12 h intervals by placing flies in empty vials with moist foam stoppers in a water bath. In between heat shocks flies were kept on yeast at 25°C. Following heat shock treatments flies were mated to wild type flies and kept well fed at 25°C for a time course starting with the last heat shock.

### Dissection and staining

All dissections were done in Schneider's cell culture medium (Life Technologies, Paisley, UK) at room temperature (RT) for no longer than 15 min before fixation in 4% (w/v) Paraformaldehyde/PBS/50 mM EDTA, pH 7.0 for 10 min. After fixation tissue was at RT rinsed in PBS, 0.1% (w/w) Triton X-100 (PBTx), extracted with 1% (w/w) Triton X-100 for 30 min and blocked with PBTx, 10% (v/v) goat serum for at least 30 min. Staining was done in blocking solution at 4°C over night (primary antibodies) or at RT for 2 h (secondary antibodies). Primary antibodies were affinity purified rabbit anti BALL (1:400, generated against residues 1–352 of BALL, A.H.), rabbit anti VASA (1:2500, generated against full length Vasa, A.H.), rabbit anti Cleaved Caspase3 Asp175 (Xu et al., 2006) (1:150, Cell Signaling Technologies, Boston, MA), rabbit anti pSMAD1 PS1 (1:500, gift from C. H. Heldin, Uppsala, Sweden), rabbit anti GFP (1:500; Synaptic Systems, Göttingen, Germany), mouse anti HTS 1B1 (1:10, DSHB, Iowa, USA), mouse anti ORB (4H8/6H4 1:1 mix, diluted 1:30, DSHB), mouse anti SHG DCAD2 (1:10), mouse anti Fibrillarin 38F3 (1:1000, Abcam, Cambridge, UK), rat anti BAM-C (1:500) (McKearin and Ohlstein, 1995), chicken anti beta-Galactosidase (1:1000, Abcam). Secondary antibodies against mouse and rabbit IgGs were coupled to Alexa-488, −568, −633 (1:400, Life Technologies). Secondary antibodies against chicken IgY were coupled to Cy5 (1:400, Abcam). After antibody incubations tissue was rinsed twice in PBTx and washed 3 times for 20 min in PBTx at RT. To visualize actin, staining with Phalloidin coupled to Alexa 568 (Life Technologies) was done at 2 U/ml in PBTx for 30 min at RT. For staining DNA, tissue was treated with RNaseA at 2 mg/ml in PBTx for 30 min, followed by staining with 10 µM draq5 (Biostatus, Shepshed, UK) or 1 µg/ml propidium iodide (Life Technologies) in PBTx for 10 min, respectively. Before mounting in Prolong Gold antifade-medium (Life Technologies) tissue was rinsed and washed for 10 min in PBTx. For identification of GFP-marked clones direct GFP fluorescence was assayed. For analysis of the *bam-GFP* reporter expression germaria were stained with anti GFP primary and Alexa 488 coupled secondary antibodies.

### rDNA FISH for STED microscopy

Germaria were fixed 4 min at 37°C in 4% paraformaldehyde, 15 mM PIPES, 80 mM KCl, 20 mM NaCl, 2 mM EDTA, 0.5 mM EGTA, 0.5 mM spermidine, 0.15 mM spermine, 1 mM DTT pH 7.2, rinsed 4 times with 2× SSC, 0.1% Tween-20 (WB), washed 10 min at RT in WB and incubated 30 min at RT with 1 mg/ml RNase A (DNase free, Sigma). After two washes with WB for 10 min at RT, germaria were rinsed and washed once in 2× SSC, 30% formamide (deionized, Sigma), 0.1% Tween-20 (PHB) at RT. After incubation with new PHB at 37°C for 30 min PHB was removed and 60 µl of probe solution was added that contained a 1 µM mix of 5′ biotinylated oligonucleotides in 3× SSC, 30% formamide, 0.1% Tween-20 (HYB). Oligonucleotides were (5′ to 3′): taaagaattttatcaagagt, caaacacctcgtcattaac, aggcagtggttgccgacctc, atattgttcaaaacgtatgt, catatgattttggcaattatatgag, aattaaatcatatacatatgaa, atatttattatatgtataagtg, aatattgaaatattcccatattctc, gtattatagagaatataattaat. Germaria were incubated with the probe solution for 15 min at 37°C, heated for 2 min to 91°C and incubated over night at 37°C. Then 500 µl of PHB were added at 37°C and germaria were washed in PHB 4 times for 10 min at 37°C.

For detection germaria were washed once in EB and twice in WB for 20 min at RT each and then for 20 min in blocking reagent/WB (Roche). Then Streptavidin-dye conjugate SA-Atto647N (ATTO-TEC, Siegen, Germany) diluted 1:25 in 250 µl new blocking solution was added. Incubation was for 2 h at RT in a thermomixer at 900 rpm. Germaria were then rinsed once and washed three times for 20 min at RT in WB. DNA was counterstained with 10 µM draq5 (Biostatus) in WB and samples were mounted in Prolong Gold antifade medium (Invitrogen). Images were acquired on a Leica TCS SP8 STED microscope.

### Image data collection and processing

For nucleolar size measurements z-stacks were collected at 0.1 µm z intervals and analysed by a modified Connected Threshold Grower plugin for Image J. Details on the modified plugin are available on request.

## Supplementary Material

Supplementary Material
