# Supplementary material for: Bällchen is required for self-renewal of germline stem cells in Drosophila melanogaster
Source: Biol Open. 2014 May 29;3(6):510–21. doi: 10.1242/bio.20147690 (PMC4058086; doi:10.1242/bio.20147690)
Supplement: Supplementary Material [file supp_3_6_510__index.html]

Bällchen is required for self-renewal of germline stem cells in Drosophila melanogaster — Bällchen is required for self-renewal of germline stem cells in Drosophila melanogaster — Supplementary Material 

# Bällchen is required for self-renewal of germline stem cells in *Drosophila melanogaster*

## bio.20147690 Supplementary Material

**Files in this Data Supplement:**

- Supplementary Material - Bettina Herzig et al. doi: 10.1242/bio.20147690
