# Supplementary material for: Bällchen is required for self-renewal of germline stem cells in Drosophila melanogaster
Source: Biol Open. 2014 May 29;3(6):510–21. doi: 10.1242/bio.20147690 (PMC4058086; doi:10.1242/bio.20147690)
Supplement: Supplementary Material [file supp_bio.20147690_bio.20147690-s1.pdf]

Biology Open (2014) 000, 1–12 doi:10.1242/bio.20147690

Supplementary Material

Bettina Herzig et al. doi: 10.1242/bio.20147690

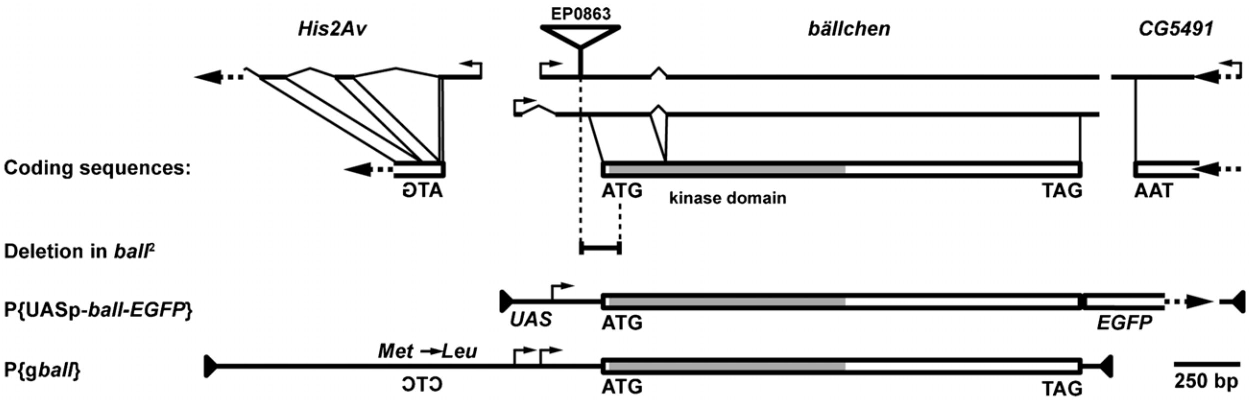

Fig. S1. Schematics of the *ball* locus, the *ball*^{2} mutation and *ball* transgenes used in this study. Schematic illustration of the *ball* locus, and the adjacent genes His2Av and CG5491, which are transcribed in opposite orientation relative to *ball*. Transcription start sites are indicated by orthogonal arrows, introns by angled lines, coding sequences by boxes and dashed line arrows indicate that not the entire sequence is displayed. Two alternatively spliced transcripts are transcribed from the *ball* locus which both code for an identical protein. We originally identified the *ball* gene based on the male sterile phenotype associated with the EP(3)0863 P-element integration (EP0863) into the *ball* 5′UTR (*ball*^{1}). By mobilization of EP(3)0863 we could revert male sterility and recovered the wild type allele *ball*^{rev}. Imprecise excision of EP(3)0863 led to partial deletion of the P-element and an associated 152 base pair deletion in the *ball* 5′UTR and coding sequence (*ball*^{2}). The deletion associated with the *ball*^{2} allele removes the initiation codon and parts of the BALL kinase domain. The *ball*^{2} allele is lethal either homozygous or if hemizygous over the deficiencies Df(3R)ro80b and Df(3R)Tl-I but not over the deficiency Df(3R)Tl-X or the amorphic His2Av^{810} allele. To rescue the *ball*^{2} mutant phenotype we constructed a GAL4 inducible transgene for expression of a BALL-EGFP fusion protein (P{*UASpball-EGFP*}). In addition we constructed a 3.5 kb genomic transgene including 1.5 kb of sequence 5′ to the *ball* initiation codon and 218 bp of sequence 3′ to the termination codon (P{g*ball*}). The intron within the *ball* coding sequence was omitted. Within P{g*ball*} the initiation codon of His2Av was changed from ATG to CTC, replacing the only Met in His2Av by Leu. The P{g*ball*} transgene rescued homozygous *ball*^{2} mutants to viability and fertility. Based on these observations we concluded that the *ball*^{2} allele does not affect other essential genes.

Biology Open (2014) 000, 1–12 doi:10.1242/bio.20147690

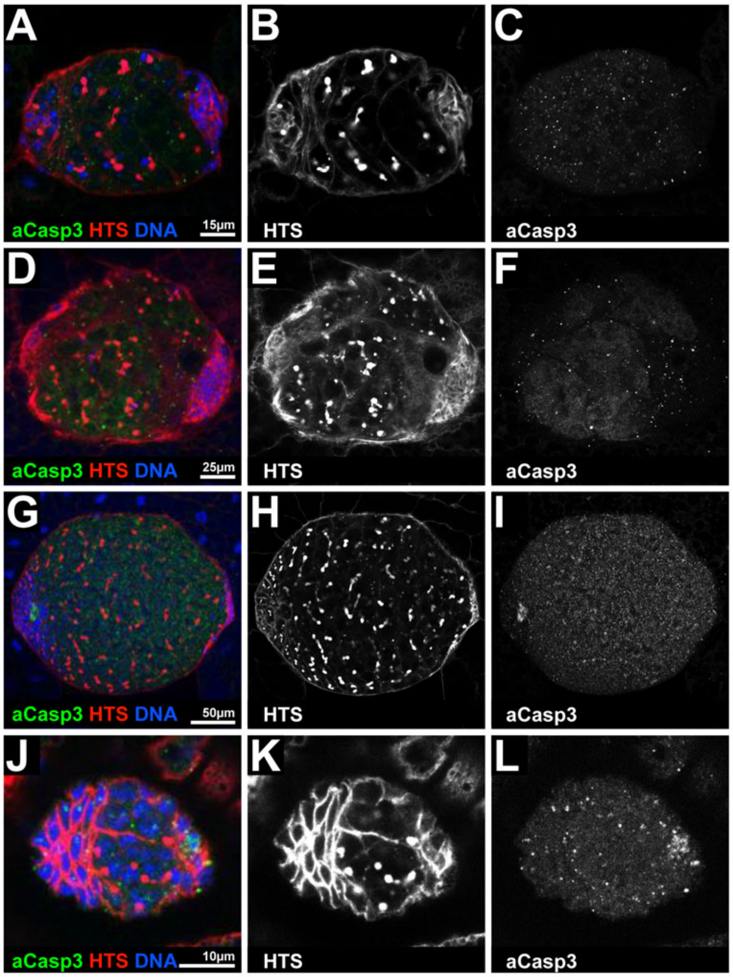

Fig. S2. Staining for apoptotic cells in *ball*^{2} mutant larval gonads. Gonads of *ball*^{2} mutant larvae were stained for the apoptosis marker activated Caspase 3 (aCasp3), HTS and DNA. (A–C) Mutant testis at mid larval stage (48 h ALH) did not show an increase in apoptotic cells. (D–F) Also at late larval stage (96 h ALH), no increase in apoptosis was detectable. (G–I) Wild type testis at late larval stage (96 h ALH) shown for comparison and to illustrate that our stainings were able to detect occasionally appearing apoptotic cells in wild type. (J–L) Mutant ovaries at mid larval stage (48 h ALH) show no apoptotic cells. Anterior is to the left in the micrographs. Scale bars: 15 μm (A–C), 25 μm (D–F), 50 μm (G–I), 10 μm (J–L).

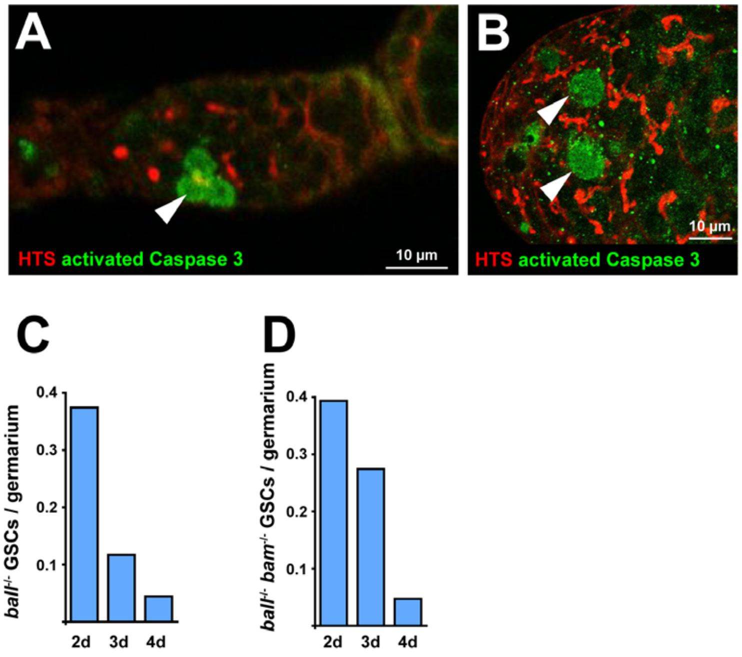

Fig. S3. Staining for apoptotic cells in *ball*^{2} mutant adult gonads. Germaria and testis were stained for HTS and with an antibody detecting cleaved, activated Caspase 3. (A,B) Confocal sections are shown to illustrate that apoptotic cells were detectable in our stainings of wild type germaria (A) or testis (B). (C) Upon induction of *ball*^{2} mutant clones, germaria were analyzed after two days (n=86), three days (n=115) and four days (n=70) for activated Caspase 3 staining. Neither mutant GSCs nor mutant CBs were found to be apoptotic, although we could reproduce the loss of GSCs over the time-course. (D) Upon induction of *ball*^{2} *bam*$^{\Delta 86}$ mutant clones, germaria were analyzed after two days (n=92), three days (n=87) and four days (n=78) for activated Caspase 3 staining. No mutant cells were found in the stem cell niche. Therefore the loss of niche associated GSCs that we observed during the time-course is not caused by apoptotic cell death. Anterior is to the left in the micrographs. Scale bars: 10 μm.

Biology Open

Biology Open (2014) 000, 1–12 doi:10.1242/bio.20147690

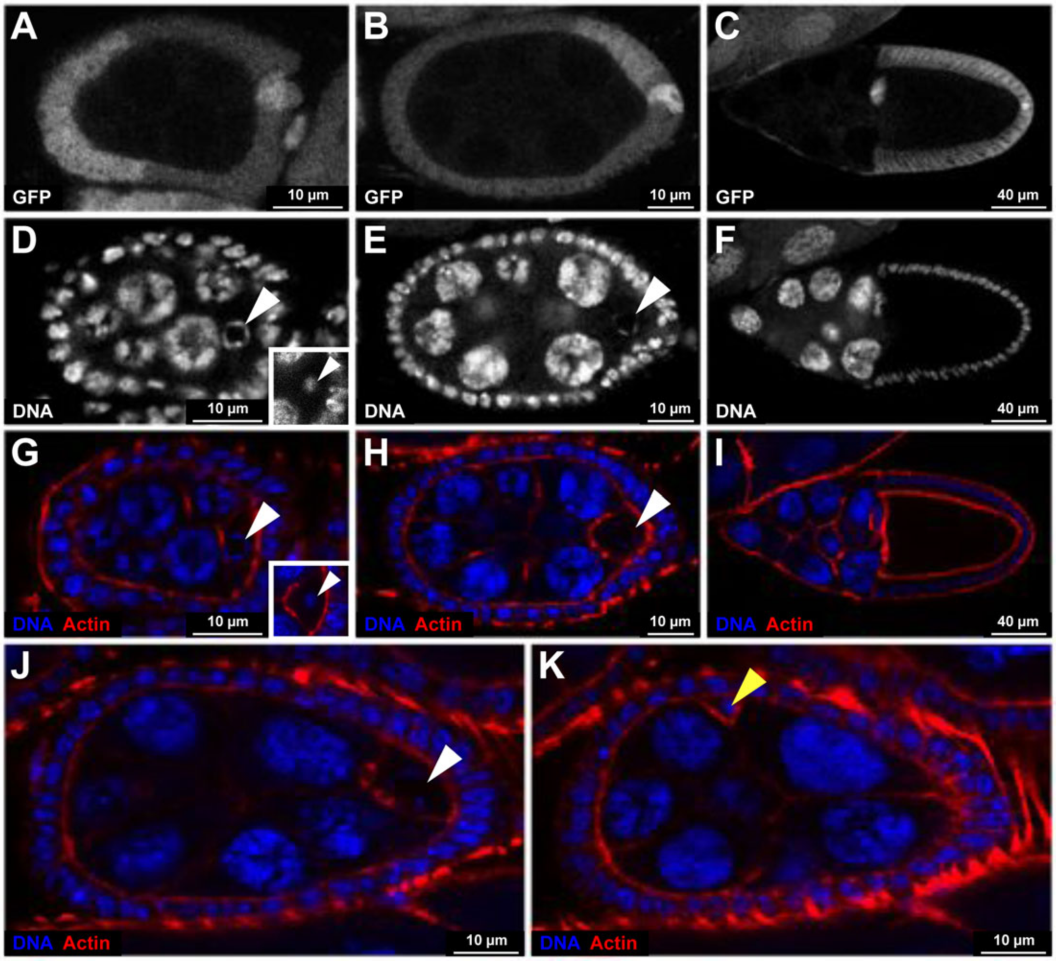

Fig. S4. Proliferation of *ball*^{2} mutant germline cyst cells. (A–I) Marked *ball*^{2} mutant germline clones were identified by reduced GFP signal (note the GFP signal in follicle cells). Egg chambers were counterstained for DNA and Actin. All images represent confocal sections. Examples are shown for stage 4 (A,D,G), stage 5 (B,E,H) and stage 10A (C,F,I) egg chambers. Most aspects of egg chamber development, including polyploidization of nurse cells, appeared to be normal in *ball*^{2} mutant egg chambers. We never observed degenerating mutant egg chambers. In all mutant egg chambers we found a defect in oocyte nuclear organization (white arrowheads, inset in panels D and G shows wild type oocyte nucleus). (J,K) Two confocal sections of a stage 7 egg chamber with a *ball*^{2} mutant germline and a defective oocyte nucleus (J, white arrowhead). In a different section a nurse cell nucleus that failed to endoreduplicate is indicated (K, yellow arrowhead). Anterior is to the left in the micrographs. Scale bars: 10 µm (A,B,D,E,G,H,J,K), 40 µm (C,F,I).

Biology Open

Biology Open (2014) 000, 1–12 doi:10.1242/bio.20147690

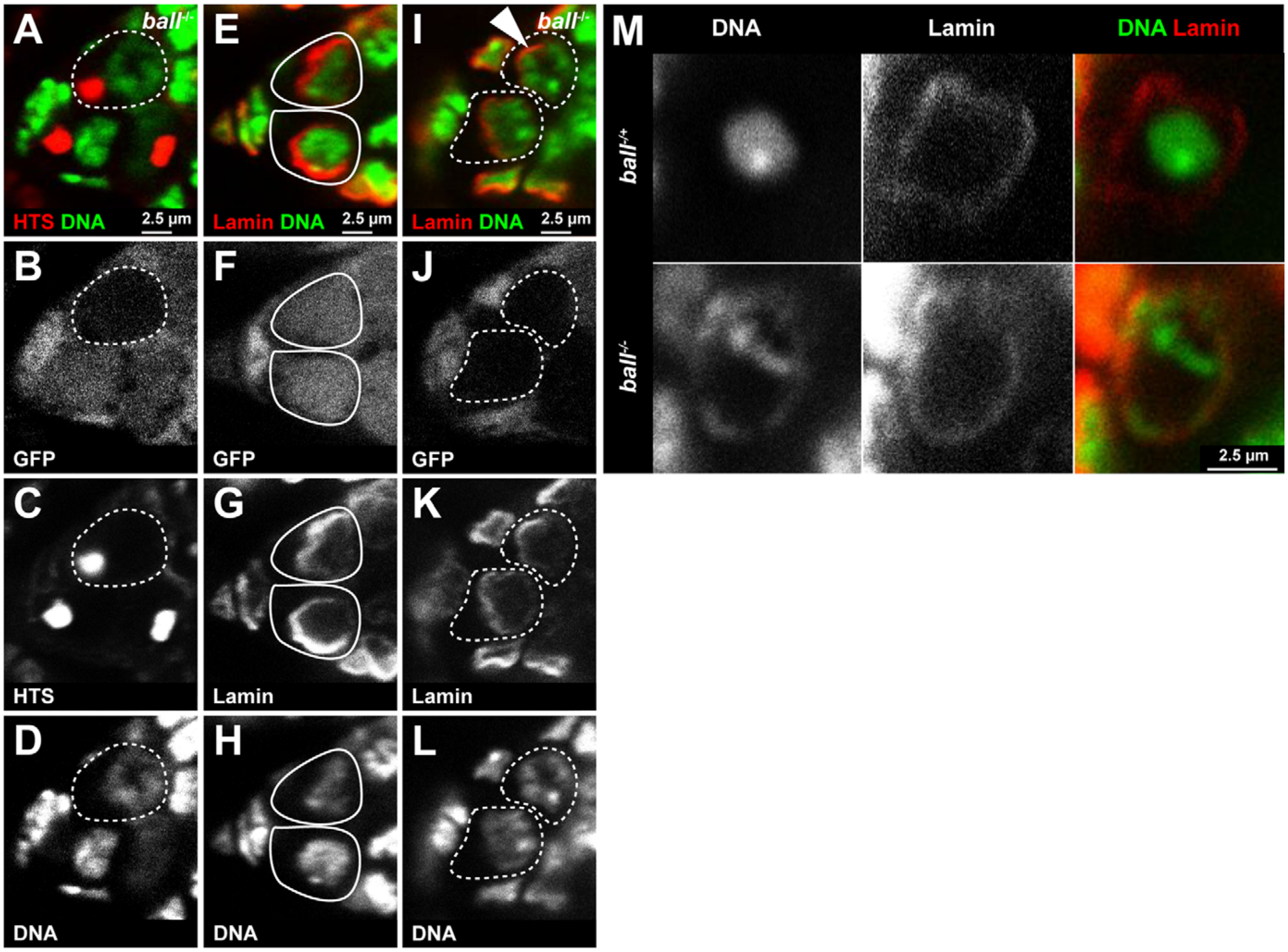

Fig. S5. Chromatin organization defects in *ball*^{2} mutant GSCs. Marked *ball*^{2} mutant cells were identified by reduced GFP fluorescence. Preparations were counterstained for DNA and either HTS to visualize spectrosomes, or for the B-type Lamin Dm0 (Lamin). Images represent confocal sections. (A–D) Example of a *ball*^{2} mutant GSC (dashed outline) indicating that loss of BALL activity leads to mild defects in nuclear organization in GSCs. In this mutant GSC chromatin is preferentially localized at the nuclear periphery leading to depletion of chromatin from the central nucleus. (E–H) Germarial tip showing two heterozygous GSCs (solid outlines). (I–L) Germarial tip showing two *ball*^{2} mutant GSCs of which one shows abnormal nuclear organization (arrowhead). In the second GSC, the effect is less pronounced. Counterstaining for Lamin revealed that assembly of the nuclear Lamina is not perturbed in *ball*^{2} mutant GSCs. (M) Oocyte nuclei from heterozygous (*ball*$^{-/+}$) or *ball*^{2} mutant (*ball*$^{-/-}$) germline clones. Defective karyosome formation in *ball*^{2} mutant oocytes is accompanied by extensive re-localization of chromatin to the nuclear periphery, which is marked by Lamin staining. Anterior is to the left in the micrographs. Scale bars: 2.5 µm.
